# Portable Electrochemical System and Platform with Point-of-Care Determination of Urine Albumin-to-Creatinine Ratio to Evaluate Chronic Kidney Disease and Cardiorenal Syndrome

**DOI:** 10.3390/bios14100463

**Published:** 2024-09-27

**Authors:** Shuenn-Yuh Lee, Ding-Siang Ciou, Hao-Yun Lee, Ju-Yi Chen, Yi-Chieh Wei, Meng-Dar Shieh

**Affiliations:** 1 Department of Electrical Engineering, National Cheng Kung University, Tainan 701401, Taiwan; gary4842002@yahoo.com.tw (D.-S.C.); lhyjason2004@yahoo.com.tw (H.-Y.L.); 2 Department of Internal Medicine, National Cheng Kung University Hospital, College of Medicine, National Cheng Kung University, Tainan 701401, Taiwan; 3 Department of Industrial Design, National Cheng Kung University, Tainan 701401, Taiwan; ejw1059@gmail.com (Y.-C.W.); mdshieh@mail.ncku.edu.tw (M.-D.S.)

**Keywords:** electrochemical sensor, chronic kidney disease, point-of-care testing, urine albumin-to-creatinine ratio, electrochemical system and platform

## Abstract

**Abstract:** The urine albumin (Alb)-to-creatinine (Crn) ratio (UACR) is a sensitive and early indicator of chronic kidney disease (CKD) and cardiorenal syndrome. This study developed a portable and wireless electrochemical-sensing platform for the sensitive and accurate determination of UACR. The developed platform consists of a carbon nanotube (CNT)-2,2′-azino-bis(3-ethylbenzothiazoline-6-sulphonic acid)(ABTS)-based modified UACR sensor, a miniaturised potentiostat, a cup holder embedded with a magnetic stirrer and a smartphone app. The UACR sensing electrode is composed of two screen-printed carbon working electrodes, one screen-printed carbon counter electrode and a screen-printed AgCl reference electrode. The miniaturised potentiostat, which is controlled by the developed app, performs cyclic voltammetry and amperometry to detect Alb and Crn, respectively. Clinical trials of the proposed system by using spot urine samples from 30 diabetic patients indicate that it can accurately classify all three CKD risk statuses within 30 min. The high accuracy of our proposed sensing system exhibits satisfactory agreement with the commercial biochemical analyser TBA-25FR (Y = 0.999X, R^2^ = 0.995). The proposed UACR sensing system offers a convenient, reliable and affordable solution for personal mobile health monitoring and point-of-care urinalysis.

## 1. Introduction

Chronic kidney disease (CKD) is amongst the most prominent causes of mortality, projected to become the fifth leading cause of death by 2040 [1] and the fastest-growing disease burden globally, according to the Global Burden of Disease Study [2]. The estimated global prevalence of the five stages of CKD is approximately 13.4% [3], corresponding to over one billion individuals worldwide. During the early stage of CKD, only a few subclinical symptoms can be observed, and awareness of CKD remains low; only one-third of patients, or even lower, are conscious of the disease [4]. In addition, CKD is considered an accelerator of cardiovascular disease (CVD) risk and an independent risk factor for CVD events [5]. Mild to moderate CKD is strongly associated with increased CVD morbidity and mortality [6]. Accordingly, clinical interventions during the early stages of CKD may not only delay or halt CKD progression but also prevent CVD outcomes. CKD patients are more likely to die from CVD events than end-stage renal failure. Early intervention in CKD is effective in reducing the high burden of related costs [7]. This effectiveness underscores the urgent and substantial unmet need, particularly in developing countries, for effective and affordable early CKD detection methods.

CKD is diagnosed by architectural kidney damage (abnormal albuminuria) and reduced renal function (estimated glomerular filtration rate) [8]. Albuminuria is accessed through 24 h urine albumin (Alb) excretion in the golden standard [9], which is cumbersome, time-consuming and associated with poor compliance in clinical practice [10]. Meanwhile, the urinary Alb-to-creatinine (Crn) ratio (UACR) calculated from a random urine sample is highly correlated with the 24 h urine Alb excretion [11] and is regarded as the most effective and convenient method for evaluating abnormal albuminuria [12,13]. UACR is not only a significant biomarker for a CKD diagnosis (<30 mg/g as normal, 30 to 300 mg/g as moderate and >300 mg/g as severe albuminuria) [14] but also a notable risk factor for predicting cardiorenal syndrome [15,16] and subtype stoke [17] in low-grade albuminuria (10–30 mg/g). In practical applications, biochemical analysers and urine strips are commonly utilised to assess albuminuria. However, a biochemical analyser that uses immunoturbidimetry and fluoroimmunoassay to detect UACR is costly and has a high technical threshold to operate [18]. Although urine colorimetric dipsticks are convenient and low-cost, they are semiquantitative and suffer from poor sensitivity for UACR ≥30 mg/g detection (34.6–43.6%) [19]; consequently, they may misclassify samples into lower risk categories and are unsuitable for an early CKD diagnosis that corresponds to a direct effect on health and survival.

Portable devices integrated with electrochemical sensing programmes have been utilised to detect biomarkers in body fluids and are widely used in personal mobile health and point-of-care (POC) monitoring [20,21]. The current study developed a promising approach for urinalysis to detect UACR, characterised by low cost, easy preservation and high accuracy, by integrating an electrochemical sensing system and platform. At present, the previously carbon nanotube (CNT)-2,2′-azino-bis(3-ethylbenzothiazoline-6-sulphonic acid)(ABTS)-based modified Alb [22] and Crn [23] sensors have been improved and evolved into one enzyme-like UACR sensing system and platform suitable for practical POC applications. For this purpose, the following statements outline the advantages and improvements of this UACR sensor compared with the previous version. The Alb and Crn sensing processes are designed for simultaneous and automatic detection. The sensing characteristics of urinary Alb and Crn have been enhanced by establishing calibration curves in urine. The accuracy of the Crn sensor is improved by sensing and subtracting the offset current at the beginning of UACR detection by using the developed electrochemical system. The combined preprocessing and detection time for the Alb sensor is reduced from 2 h to 30 min. Urine samples from 30 diabetic patients are tested to confirm the feasibility of the presented UACR detection system and platform. This approach demonstrates the remarkable potential for the POC monitoring and the diagnosis of UACR, providing results that are nearly comparable with those of commercial biochemical analysers.

## 2. Materials and Methods

### 2.1. Chemicals and Materials

Chemicals used for this work include Na_2_SO_4_, Na_2_HPO_4_, creatinine, human serum albumin, multi-walled CNT, ABTS, 0.5 M hydrochloric acid solution, and 5 wt. % nafion perfluorinated resin solution; purchased from Sigma-Aldrich, St. Louis, MI, USA, and utilised as received without further purification. Customised electrodes (Zensor R&D, Taichung City, Taiwan) were fabricated using a five-layer manufacturing process (polypropylene [PP] cutting, Ag printing, AgCl printing, carbon printing, and insulator covering) and used as received. All electrical devices (Analog Devices, Wilmington, MA, USA) used in the proposed electrochemical module were soldered on a printed circuit board (PCB) and powered by a 3.3 V battery. The primary instruments included an electrochemical analyser (660e, CH Instruments, Bee Cave, TX, USA) and an automatic biochemical analyser equipment (TBA-25FR, Canon Medical Systems, Ōtawara, Japan) for the correlation of the urinalysis results. All calibrators and quality control reagents used for Crn and Alb detection on the TBA-25FR were purchased from Tunyen Enterprise Corporation. Deionized water was used for the preparation of the phosphate-buffered solution (PBS) and the dispersed CNT-ABTS solution.

### 2.2. Manufacture of the Customised Electrode

**Figure 1:**
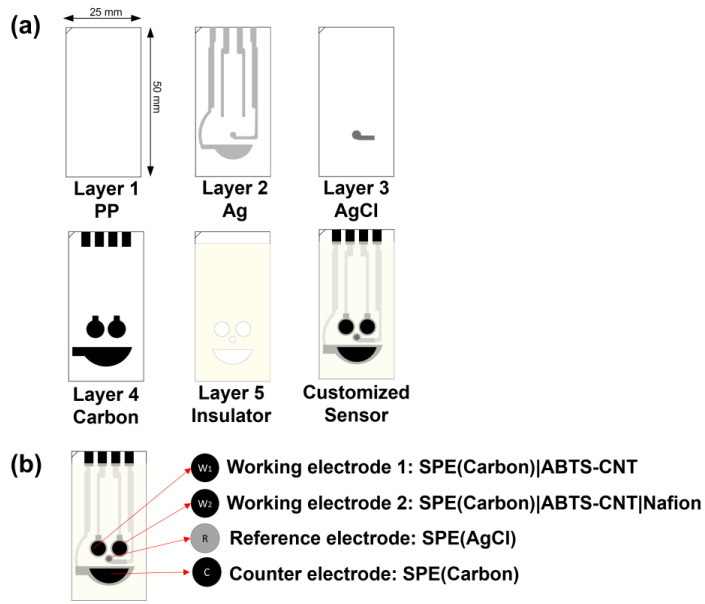
(**a**) Layered graph of the customised electrode. (**b**) Proposed electrochemical UACR dual working sensor.

The customised electrode was fabricated through a five-layer manufacturing process, with overall dimensions of 25 mm × 50 mm × 0.5 mm (Figure 1a). The first PP layer, with a thickness of 0.5 mm, served as the base of the electrode and was selected for printing Ag, AgCl and carbon. The second Ag layer was designed to transmit electric current for the three-electrode system. The AgCl reference electrode (RE) was printed on the third layer. The fourth carbon layer consisted of two circles (ø = 5.00 mm), which were screen-printed electrodes (SPE) that functioned as the dual working electrodes (WEs). The half-oval one was the counter electrode (CE), and four pins connected the electrochemical system. The final layer was the photocuring-printed ultraviolet ray resin (PICS-Isolation Ink-U001), which functioned as the insulator.

### 2.3. Design of the Proposed Electrochemical System

**Figure 2:**
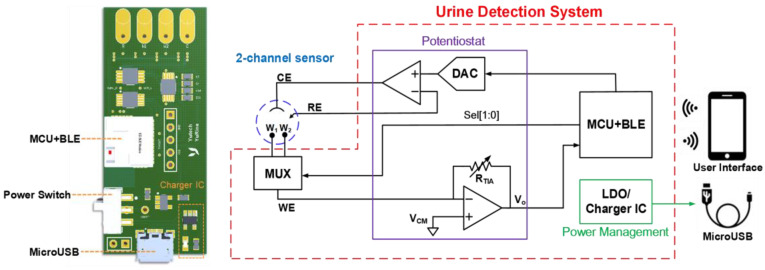
Proposed electrochemical detection system.

Figure 2 illustrates the proposed electrochemical system, which consists of a 2:1 multiplexer (MUX), a reliable potentiostat circuit, a microcontroller unit (MCU) with a Bluetooth Low Energy (BLE) module (Infineon Technologies CYBLE-222014-01, Neubiberg, Germany), a power management circuit with a universal serial bus (USB) charger and a customised smart application platform (app) as the user interface. The core of the electrochemical readout circuit is the potentiostat, which can provide redox reaction and signal acquisition with two operational amplifiers (Analog Devices AD8607ARMZ, Wilmington, MA, USA). In a three-electrode electrochemical measurement, the conventional approach for detecting sensor currents is utilising a trans-impedance amplifier (TIA) to amplify the weak redox reaction current for signal acquisition with a high-resolution analog-to-digital converter (ADC). The potentiostat can provide the chronoamperometry (CA) and cyclic voltammetry (CV) programmes for detecting Crn and Alb, respectively. The MUX (Analog Devices ADG839YKSZ, Wilmington, MA, USA), which is controlled by the MCU in the BLE module, enables two different electrochemistry of the dual WEs to be reacted consecutively, following the programme of detection progress. The potentiostat circuit is directed by the MCU to determine which wave pattern (step or triangle wave) to output from the dual-channel digital-to-analog converter (DAC) (Analog Devices AD5623RBRMZ, Wilmington, MA, USA) and automatically operate the experiments. The power management circuit is composed of a 3.3 V low-dropout regulator (Analog Devices ADP151AUJZ-3.3, Wilmington, MA, USA), providing the electrochemical system with a stable 3.3 V input voltage and a charge integrated circuit (Analog Devices LTC4054ES5-4.2, Wilmington, MA, USA) connected to a USB connector (Amphenol FCI 10118193-0001LF, Bangalore, India) for charging the rechargeable lithium-ion battery.

**Figure 3:**
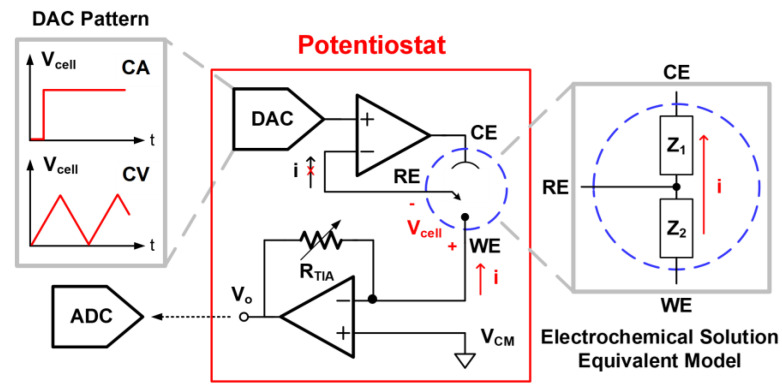
Design of the potentiostat for performing the CA and CV programmes.

Figure 3 depicts the design of the potentiostat for the CA and CV programmes. The WE is regarded as an electronic provider or receiver under different DAC patterns of V_cell_. In CA measurement, the MCU directs the DAC to provide the step wave to the RE, producing a constant potential difference across the WE and RE. The CE works as the current source to provide a current path to the WE, adopting an operational amplifier as the output driver. The mechanism is similar to a non-inverting amplifier operation with an effective feedback network of urine impedance between WE/RE/CE. The RE remains at a fixed potential throughout the reaction in this pattern. In the CV mode, the MCU directs the DAC to implement the triangle wave, causing the varying potential difference at a programmable scan rate. The equivalent impedance model of the electrochemical solution can be described as Z_1_ (CE to RE) and Z_2_ (RE to WE). The oxidation or redox current goes through the WE and CE, and no current passes through the RE due to the high input impedance of the operational amplifier through the virtual ground. The reaction current is converted into the voltage domain as an output node of V_o_ with the TIA. This voltage signal will be quantised by the ADC in the MCU module for further calculation. After signal acquisition, the ADC output signal is transmitted to the smartphone app through BLE. Accordingly, the analyte concentration will be calculated from the ADC output and displayed on our customised user app.

This dual-channel electrochemical urine detection system is developed into a small PCB to be a portable device (Figure 2). The size of the device is only 50 mm × 21.59 mm; thus, it can be easily integrated into our prototype device. Urine detection can be simply executed with the highly integrated PCB and the customised user platform; hence, the device is useful either for personal health administration or home-care systems.

### 2.4. Preparation of the UACR Sensor

A dispersed 2,2′-azino-bis(3-ethylbenzothiazoline-6-sulphonic acid) (ABTS)–carbon nanotube (CNT) solution was prepared by dispersing CNT (3 mg mL^−1^) in ABTS that contained an aqueous solution (6 mg mL^−1^) under ultrasonication for at least 30 min in advance. WEs 1 (W_1_) and 2 (W_2_) were modified by drop-casting an aliquot of 10 μL of the prepared CNT-ABTS dispersion solution (Figure 1b). After being dried at room temperature, the SPE(W_1_)|CNT-ABTS electrode was used for Alb detection. The Crn sensor (W_2_) was further modified with a layer of Nafion film by drop-casting a 10 μL aliquot of Nafion solution (5%), designated as SPE(W_2_)|CNT-ABTS|Nafion. The RE and CE were used as received.

### 2.5. Characterisations of the UACR Sensing System and Platform

**Figure 4:**
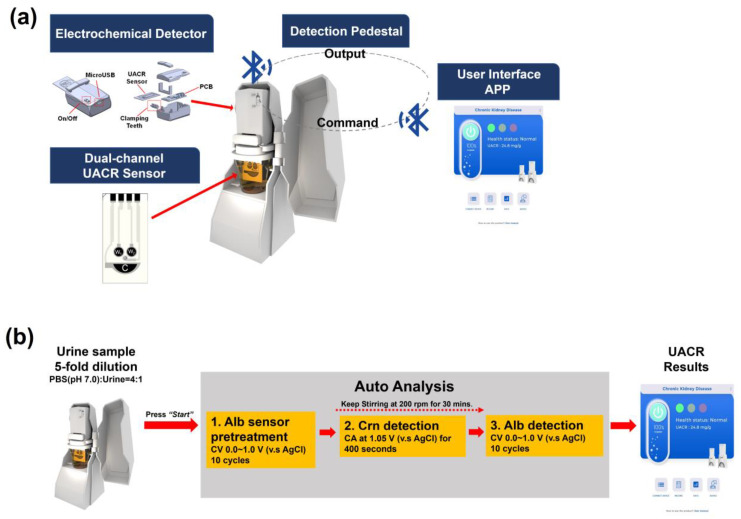
(**a**) Proposed portable UACR electrochemical system and platform. (**b**) UACR examination process of the proposed portable electrochemical system and platform.

Figure 4a illustrates the proposed sensing system, which consists of a disposable UACR sensing electrode, a portable electrochemical detector, a cup holder embedded with a magnetic stirrer, and the developed app. Figure 4b shows the process of UACR examination by the proposed system and platform. User operation time is less than 1 min due to autoanalysis. Urine samples were collected from diabetic patients in the National Cheng Kung University Hospital. Amperometric Crn detection and voltammetric Alb detection in real urine samples were performed using SPE(W_2_)|CNT-ABTS|Nafion and SPE(W_1_)|CNT-ABTS_(CV)_ electrodes, respectively. The test samples were prepared by adding 5 mL of urine to 20 mL of 0.1 M PBS (pH 7.0) using a 5 mL pipette into a 30 mL cup. The stirring process controlled by wireless BLE was achieved through the magnetic stirrer inside the cup holder.

Initially, the UACR sensor was connected to the powered-on electrochemical detector and immersed in the prepared test sample. The user app and the electrochemical detector were then connected via wireless BLE. After pressing the start button, the electrochemical detector and magnetic stirrer carried out the UACR detection program in four automatic measurement steps:

(i) The Alb sensor pretreatment was performed with CV analyses (potential range: 0–1.0 V versus Ag/AgCl; scan rate: 100 mV s^−1^; cycles: 10), after which W_1_ is represented as SPE(W_1_)|CNT-ABTS_(CV)_.
(ii) After the Alb sensor pretreatment, magnetic stirring was initiated at 200 rpm for 30 min. The Crn sensor (W_2_) performed amperometric detection for 400 s at 1.05 V versus Ag/AgCl in the stirred sample. The current density at the end of the amperometric detection was recorded, and the Crn concentration was then calculated using the calibration curve of SPE(W_2_)|CNT-ABTS|Nafion with the developed app.
(iii) By the end of the 30 min magnetic stirring, CV analyses (potential range: 0–1.0 V versus Ag/AgCl; scan rate: 100 mV s^−1^; cycles: 10) were applied on the SPE(W_1_)|CNT-ABTS_(CV)_. The Alb concentration was then calculated using the calibration curve of SPE(W_1_)|CNT-ABTS_(CV)_ with the developed app.
(iv) The UACR results calculated from the values of Crn and Alb and the corresponding risk levels by the clinically relevant CKD diagnostic criteria were displayed on the customised user app.

## 3. Results and Discussion

### 3.1. Alb and Crn Reaction Setting

In accordance with our team’s previous studies [22,23,24], the detection of Crn based on ABTS and Crn chemical reaction was performed at an optimal incubation pH of 7.0, with stirring at 200 rpm for 400 s. Alb sensing was based on the electrochemical oxidation of the amino acid residues of Alb at pH 9.0, with 200 rpm stirring for 30 min. In previous studies, the optimal pH for Alb detection was set to 9.0. However, considering the pH range of urine samples (5.5–7.5 [25]) and the integration of both sensors in the UACR experiment, we chose pH 7.0 as the incubation solution for the detection of both Crn and Alb.

Due to the effects of biofluids [26,27], such as ionic strength, pH value, or conductivity, which can influence electrochemical reactions, the calibration curves were established under the same conditions as those used in practical clinical trial applications. Test samples were prepared by adding 5 mL of urine to 20 mL of PBS (0.1 M, pH 7.0) to stabilize the incubation solution at pH 7.0 and electrolyte conductivity. The benchmark values of Crn and Alb in the urine were measured using a biochemical analyzer (TBA-25FR) for the establishment and validation of the calibration curve.

### 3.2. Voltammetric Alb Detection by SPE(W_1_)|CNT-ABTS

**Figure 5:**
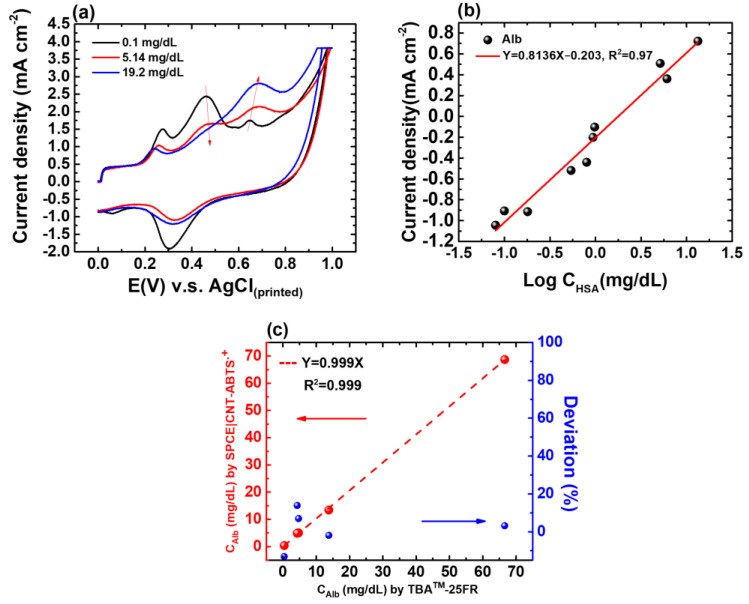
(**a**) CV responses of the SPE(W_1_)|CNT-ABTS_(CV)_ to Alb of various concentrations. (**b**) Calibration curve of the proposed albumin sensor in urine with known concentrations of 0.08, 0.1, 0.18, 0.54, 0.8, 0.94, 0.98, 5.14, 6.1, and 19.2 mg/dL, respectively. (**c**) Regression analysis of C_Alb_ in five urine samples determined using TBA-25FR and the SPE(W_1_)|CNT-ABTS_(CV)_ electrode is shown in red; The deviation percentage in the C_Alb_ values determined by TBA™-25FR and SPE(W_1_)|CNT-ABTS_(CV)_ electrode is shown in blue.

Based on previous findings [22], the CNT-ABTS_(CV)_ nanozyme was functionalized to exhibit enzyme-like activity, characterized by high specificity and electrocatalytic properties, making it an exceptional recognition element for detecting Alb. In the previous study, the mechanisms behind the adsorption and electrochemical oxidation of Alb on CNT-ABTS_(CV)_ electrodes were investigated. The CNT-ABTS_(CV)_ electrodes, derived from anodic pretreatment, showed an enhanced affinity for electroactive amino acids like tryptophan (Trp) and tyrosine (Tyr). This improved affinity facilitated Alb adsorption with increased exposure of Trp and Tyr residues, boosting electrochemical oxidation activity. The adsorption of Alb on the CNT-ABTS_(CV)_ electrode involves a multisite interaction. After Alb adsorption, Alb detection can be achieved based on the measurement of the current response from the electrochemical oxidation of the adsorbed electroactive amino acids of Alb and the unlinked ABTS on the SPE(W_1_)|CNT-ABTS_(CV)_ (Figure 5a). The oxidation potential of adsorbed electroactive amino acids of Alb and the unlinked ABTS is 0.69 V and 0.46 V versus Ag/AgCl, respectively. Noticeable increases in electroactive amino acids oxidation current and decreases in ABTS oxidation current were observed with increased concentration. The developed app will automatically identify the peaks near 0.46 V and 0.69 V versus Ag/AgCl, and then subtract the oxidation peak current of ABTS from that of Alb for the concentration calculation. Considering the diverse interactions between SPE(W_1_)|CNT-ABTS_(CV)_ and Alb, the abundant interaction sites empower high selectivity towards Alb detection.

The normal Alb level in urine is less than 20 mg/dL [28]. The pretreatment of SPE(W_1_)|CNT-ABTS was performed with a potential range of 0–1.0 V versus Ag/AgCl, a scan rate of 100 mV s^−1^, and 10 cycles, after which W_1_ is represented as SPE(W_1_)|CNT-ABTS_(CV)_. The characterizations of the Alb sensor were tested after 30 min of incubation with stirring at 200 rpm, using prepared test samples described in Section 3.1, ranging from 0.08 to 19.2 mg/dL (with initial urine Alb concentrations ranging from 0.4 mg/dL to 96.0 mg/dL before 5-fold dilution). The calibration curve of the Alb sensor is drawn by the oxidation current density of Alb minus that of ABTS on the y-axis and the logarithm of Alb concentration on the x-axis is shown in Figure 5b. The linear range is from 0.08 mg/dL to 19.2 mg/dL. The linear equation is current density(mA/cm^2^) = −0.203 + 0.8136 logC(Alb) (mg/dL). The limit of detection (LoD) is as low as 0.02 mg/dL.

To validate the calibration curve derived from test samples, five spot urine samples were analyzed using both the proposed Alb sensor and the biochemical analyzer TBA-25FR. The correlation coefficient of 0.999 (Figure 5c), obtained from the regression analysis of C_Alb_ values using both methods, demonstrated an excellent correlation between the two methods and confirmed the reliability of the proposed Alb sensor in urine sample testing. The deviation in the C_Alb_ values determined by TBA™-25FR and our proposed SPE(W_1_)|CNT-ABTS_(CV)_ electrode was less than 13.9%.

### 3.3. Amperometric Crn Detection by SPE(W_2_)|CNT-ABTS|Nafion

According to previous reports [29], the electrochemical oxidation of ABTS involves two reversible single-electron transfer steps. The first step, with a formal potential (E°’) of 0.472 V vs. Ag/AgCl, involves the oxidation of ABTS to its cation radical (ABTS^+^) (Equation (1)). The second step, with an E°’ of 0.885 V vs. Ag/AgCl, involves the oxidation of ABTS^•+^ to the ABTS dication (ABTS^2+^) (Equation (2)). Additionally, a comproportionation reaction between ABTS^2+^ and ABTS can occur during the process, forming 2 ABTS^+^ (Equation (3)). The electrochemical detection of Crn involves the electrochemical generation of ABTS^+^ (Equation (4)) at an appropriately applied potential to maximize the production of ABTS^+^. Hence, the applied potential of 1.05 V versus Ag/AgCl is chosen by our previous results [23] and hardware design.

(1) $$\mathrm{ABTS}⇄\mathrm{ABT}S^{•+}\;+e^{-}\;at\;0.472\;V\;vs.\;Ag/AgCl.$$

(2) $$\mathrm{ABTS}⇄\mathrm{ABT}S^{2+}+\;e^{-}\;at\;0.885\;V\;vs.\;Ag/AgCl.$$

(3) $$\mathrm{ABTS}+\mathrm{ABT}S^{2+}\to \;2\mathrm{ABT}S^{•+}$$

The follow-up chemical reaction between ABTS^•+^ and Crn is

(4) $$\mathrm{ABT}S^{•+}-\mathrm{Crn}\;\mathrm{chemical}\;\mathrm{reaction}:\;\mathrm{ABT}S^{•+}+\mathrm{Crn}⇄\;\mathrm{ABTS}+\mathrm{product}$$

To further assess its applicability for Crn detection in urine samples, the reactivity of various biomolecules commonly found in human urine towards the reduction of ABTS^+^ was investigated by colorimetric and amperometric methods in our previous research [23]. Colorimetric studies showed that the reduction of ABTS^+^ to ABTS occurred not only in the presence of Crn but also in the presence of other negatively charged biomolecules [30], including tyrosine, uric acid, and ascorbic acid. However, the interference from these biomolecules can be minimized significantly by incorporating a negatively charged Nafion layer into the amperometric Crn sensors. In the application for detecting Crn in urine, the SPE(W_2_)|CNT-ABTS|Nafion amperometric Crn sensor requires a 5-fold dilution of the urine samples with PBS (0.1 M, pH 7.0). This dilution helps minimize interference from other species and reduces the impact of variations in the pH of the urine samples.

**Figure 6:**
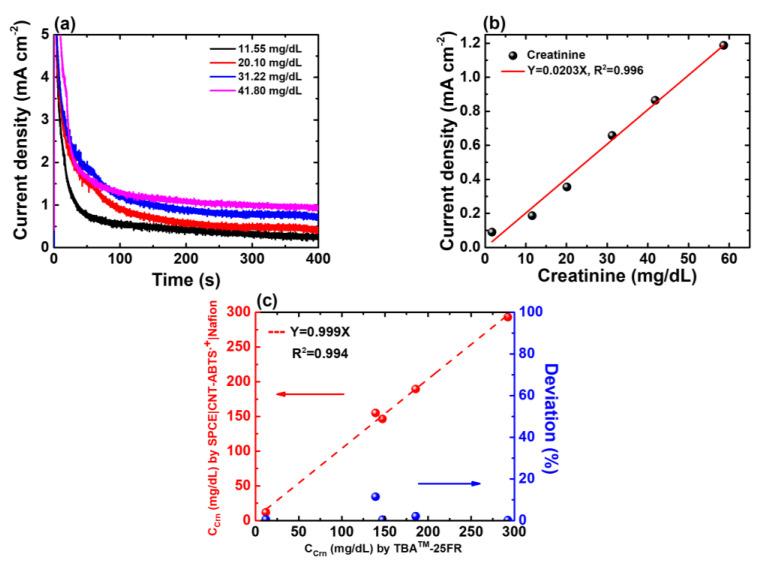
(**a**) CA responses of the SPE(W_2_)|CNT-ABTS|Nafion electrode to various Crn concentrations. (**b**) Calibration curve of the proposed Crn sensor in urine with known concentrations of 1.67, 11.55, 20.10, 31.22, 41.80 and 58.62 mg/dL, respectively. (**c**) Regression analysis of C_Crn_ in five urine samples determined by TBA-25FR and SPE(W_2_)|CNT-ABTS|Nafion is shown in red; The deviation percentage in the C_Crn_ values determined by TBA™-25FR and SPE(W_2_)|CNT-ABTS|Nafion electrode is shown in blue.

At the beginning of Crn detection, the offset current is detected and subtracted by the developed electrochemical system. After 400 s of the CA programme on SPE(W_2_)|CNT-ABTS|Nafion, the amperometric responses to the prepared Crn samples with various concentrations are presented in Figure 6a. The normal Crn level in urine ranges from 66.97 to 215.04 mg/dL [31]. The characteristics of the Crn sensor were tested using prepared test samples described in Section 3.1, ranging from 1.67 to 58.62 mg/dL (with initial urine Crn concentrations ranging from 8.36 mg/dL to 293.14 mg/dL before 5-fold dilution). The linear fitting curve of the proposed Crn sensor is shown in Figure 6b, and the linear equation is current density(mA/cm^2^) = 0.0203 C(Crn) (mg/dL) with R^2^ = 0.996. The detection limit is estimated to be 0.62 mg/dL.

To validate the calibration curve derived from test samples, five spot urine samples were collected from the hospital and analysed using the proposed Crn sensor and biochemical analyser TBA-25FR. The regression analyses determined by both methods are illustrated in Figure 6c, with a distinguished correlation coefficient (R^2^ = 0.994). It was found that the deviation in the C_Crn_ values determined by TBA™-25FR and our proposed SPE(W_2_)|CNT-ABTS|Nafion electrode was less than 11.45%.

### 3.4. Application of the Proposed Electrochemical System and Platform to UACR Test

Due to both CKD and cardiorenal syndrome being major complications associated with diabetes [32,33,34], monitoring kidney function is essential in preventing further deterioration of both renal and cardiovascular health. Therefore, diabetic patients were chosen in the clinical trial in this study. A total of 30 human urine samples were collected from diabetic patients at the National Cheng Kung University Hospital for the real sample test. The hospital provided the age and gender of the patients, and the benchmark values for Alb and Crn concentrations were determined using the TBA-25FR biochemical analyser (Table 1).

**Figure 7:**
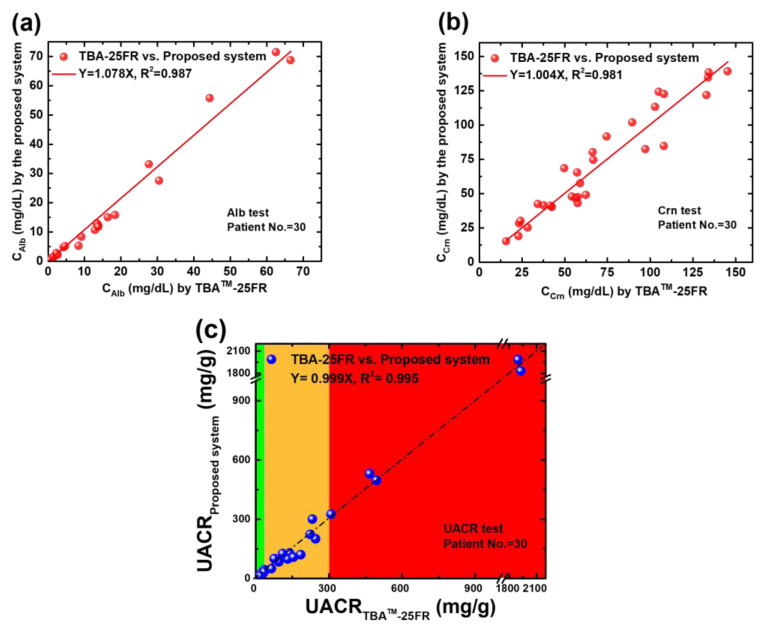
(**a**) Regression analysis of the C_Alb_ of 30 spot urine samples determined using TBA-25FR and the SPE(W_1_)|CNT-ABTS_(CV)_ electrode (**b**) Regression analysis of the C_Crn_ of 30 spot urine samples determined using TBA-25FR and the SPE(W_2_)|CNT-ABTS|Nafion electrode. (**c**) Regression analysis of the UACR value of 30 spot urine samples calculated using the Alb and Crn results of TBA-25FR and the proposed UACR sensing system and platform.

All the collected samples were tested using the proposed system and biochemical analyser. The corresponding results are presented in Figure 7. The x-axis indicates the benchmark results by TBA-25FR. The y-axis shows the experimental results obtained from the electrochemical system and platform. Good agreements can be observed between both methods (slope = 1.078 for Alb, slope = 1.004 for Crn and slope = 0.999 for UACR), with an excellent correlation coefficient in all types of tests (R^2^ = 0.987 for Alb, R^2^ = 0.981 for Crn and R^2^ = 0.995 for UACR). In the significant analysis, the correlation results between the proposed system and the biochemical analyser TBA-25FR are statistically significant (r = 0.992, *p* < 0.001 for Alb; r = 0.958, *p* < 0.001 for Crn; and r = 0.997, *p* < 0.001 for UACR) in two-tailed tests conducted using SPSS Statistics 17.0. The feasibility of the proposed electrochemical platform for UACR urinalysis is confirmed. Moreover, this platform accurately classifies all three CKD risk statuses within 30 min and successfully distinguishes between normal and low-grade albuminuria (10 mg/g to 30 mg/g) for the application of cardiorenal syndrome and stroke risk prediction, with an accuracy of 96.7%, where one low-grade albuminuria sample was misidentified as a normal sample out of 30 samples.

Table 2 compares various UACR detection systems [35,36,37,38] with the proposed system. In contrast with paper-based methods, the presented system is considerably easier to store at room temperature for months [22,23] and operates without the need for heating even though the analysis time is longer than the former. The proposed platform offers significant advantages by analysing real urine instead of artificial urine compared with a similar electrochemical method.

## 4. Conclusions

A portable, wireless, effective and affordable electrochemical system for the quantification of UACR is proposed in this study. The UACR test results of the 30 diabetic patients exhibit excellent agreement (Y = 0.999X, R^2^ = 0.995) with the benchmark values analysed using the biochemical analyser TBA-25FR, suggesting its applicability to the diagnoses of early CKD and cardiorenal syndrome or the risk prediction of subtype stroke. The presented system serves as a feasible and promising platform for practical clinical use and POC urinalysis in the future due to its promising characteristics, including auto-detection operation, low manufacturing cost (less than $0.50 USD), simple storage requirements and high accuracy.

## Figures and Tables

**Table 1:** Benchmark UACR values for the urine samples collected from diabetic patients across different genders and age groups.

Gender	Age (Years)	Number	<10 mg/g	10–30 mg/g	30–300 mg/g	>300 mg/g
Male	All	19	5	4	7	3
	<60	1	1	0	0	0
	60–80	14	3	3	5	3
	>80	4	1	1	2	0
Female	All	11	0	3	6	2
	<60	1	0	1	0	0
	60–80	5	0	1	2	2
	>80	5	0	1	4	0

**Table 2:** Comparison of several UACR sensing systems.

Sensor Configuration	Method	Analysis Time (min)	Linear Range (mg/dL)	LoD (mg/dL)	O.T. ^a^ (°C)	S.T. ^b^ (°C)	S.N. ^c^	Reference ^d^
BG ^e^/JR ^f^/PAD ^g^ s	CM ^h^	1	10–350 (Alb) 10–350 (Crn)	7.1 (Alb) 5.1 (Crn)	50	N.A. ^i^	3	[35]
CAS ^j^ −Pd^2+^ complex/drawing-PAD ^g^ s	CM ^h^	15	0-100 (Alb) 0-300 (Crn)	N.A. ^i^ N.A. ^i^	R.T. ^k^	N.A. ^i^	15	[36]
Carbon|SA ^l^ /NFC ^m^	EC ^n^	3	1–200 (Alb) 1–30 (Crn)	0.63 (Alb) 0.93 (Crn)	R.T. ^k^	N.A. ^i^	5(A.U. ^o^)	[37]
BCG ^p^ dye /PA ^q^ /sliding PAD ^g^	CM ^h^	3	0.75–10 (Alb) 10–300 (Crn)	0.5 (Alb) 5.0 (Crn)	40	−15	23	[38]
SPE|CNT-ABTS_(CV)_ SPE|CNT–ABTS|Nafion	EC ^n^	30	0.08-19.20 (Alb) 1.67-58.62 (Crn)	0.02 (Alb) 0.62 (Crn)	R.T. ^k^	R.T. ^k^	30	Present work

^a^ operation temperature; ^b^ storage temperature; ^c^ sample number; ^d^ reference; ^e^ bromocresol green; ^f^ jaffé reaction; ^g^ paper-based analytical device; ^h^ colorimetry; ^i^ not available; ^j^ chrome azurol s; ^k^ room temperature; ^l^ steric acid; ^m^ near-field communication; ^n^ electrochemistry; ^o^ artificial urine; ^p^ bromocresol green; ^q^ picric acid.

## Data Availability

The data presented in this study are available upon request from the corresponding authors.
